# On compression and damage evolution in two thermoplastics

**DOI:** 10.1098/rspa.2016.0495

**Published:** 2017-01

**Authors:** N. K. Bourne, S. C. Garcea, D. S. Eastwood, S. Parry, C. Rau, P. J. Withers, S. A. McDonald, E. N. Brown

**Affiliations:** 1 School of Materials, University of Manchester, Rutherford Appleton Laboratory, Didcot, Oxfordshire OX11 0FA, UK; 2 Diamond Light Source Ltd, Harwell Science and Innovation Campus, Didcot, Oxfordshire OX11 0DE, UK; 3 Defence Science and Technology Organisation, Adelaide, Australia; 4 Explosive Science and Shock Physics Division, Los Alamos National Laboratory, Los Alamos, NM, USA

**Keywords:** Taylor impact, thermoplastic, dynamic response

## Abstract

The well-known Taylor cylinder impact test, which follows the impact of a flat-ended cylindrical rod onto a rigid stationary anvil, is conducted over a range of impact speeds for two polymers, polytetrafluoroethylene (PTFE) and polyetheretherketone (PEEK). In previous work, experiments and a model were developed to capture the deformation behaviour of the cylinder after impact. These works showed a region in which spatial and temporal variation of both longitudinal and radial deformation provided evidence of changes in phase within the material. In this further series of experiments, this region is imaged in a range of impacted targets at the Diamond synchrotron. Further techniques were fielded to resolve compressed regions within the recovered polymer cylinders that showed a fracture zone in the impact region. The combination of macroscopic high-speed photography and three-dimensional X-ray imaging has identified the development of failure with these polymers and shown that there is no abrupt transition in behaviours but rather a continuous range of responses to competing operating mechanisms. The behaviours noted in PEEK in these polymers show critical gaps in understanding of polymer high strain-rate response.

## 1. Introduction

The adaptability of polymeric materials in function and processing has allowed manufacturers to realize new diverse applications. The ability to cast, mould or extrude them to component shapes has made plastics increasingly dominant in manufacturing [1]. Furthermore, polymers have been employed as a binder phase in composites with other material classes introduced as fibres or as embedded, second-phase particles. Their strength and flexibility has allowed them to not only be cast, but also drawn in a manner that optimizes material microstructure which benefits from the inherent strength in the polymer chain. As applications of polymers and polymer matrix composites grow, they are placed under increasingly more extreme conditions in harsh environments such as in space, or under high-temperature conditions in demanding, next-generation production environments.

The Taylor cylinder impact test is a useful integrated experiment in which a range of strain rates and flow fields upon deformation can easily be realized under relatively low velocity conditions [2–4]. Directly following impact, shock-loading defines the first, transient state in the cylinder nose, which lasts until releases intrude from its periphery [5]. During this stage loading to high pressure and subsequent releases can transform the microstructure, leading to damage sites that trigger failure at later times. Within one diameter back from the impact face, the stress attenuates so that the rest of the cylinder experiences quasi-elastic wave propagation followed by specimen deceleration. Despite this range of stresses and operating mechanisms, the behaviour (of at least the deformation of metals) was initially predicted with reasonable accuracy using an elementary mathematical model applicable to elastoplastic metals [2,6]. While the test has since been used (and analysis and models improved) extensively for metals, its use for polymers and plastics in producing new high strain-rate constitutive descriptions has proved less successful. Hutchings expanded its use into polymers to investigate the dynamic yield of polyethylene [7,8]. More recent work has employed the Taylor cylinder impact test to understand the dynamic constitutive and failure behaviour of polytetrafluoroethylene (PTFE) [9,10], polyetheretherketone (PEEK) [11–13], polychlorotrifluoroethylene [14], polycarbonate [15,16], polyethylene [17] and polyurea [18]. Material models typically only used to compare the transient sample profile/geometry and do not include failure mechanisms.

Mechanical properties of polymers are known to change dramatically with temperature, going from glass-like brittle behaviour at low temperatures to a rubber-like response at high temperatures. Polymers are also very sensitive to the rate of deformation (strain rate); indeed, increasing rate of deformation is found to have the same effect as decreasing temperature. During impact conditions we expect brittle behaviour at the impact face (where strain rates are highest) and the pressure to reduce within one diameter from there. Thus brittle damage will be localized, but increase in this zone as this impact speed increases.

**Figure 1.**
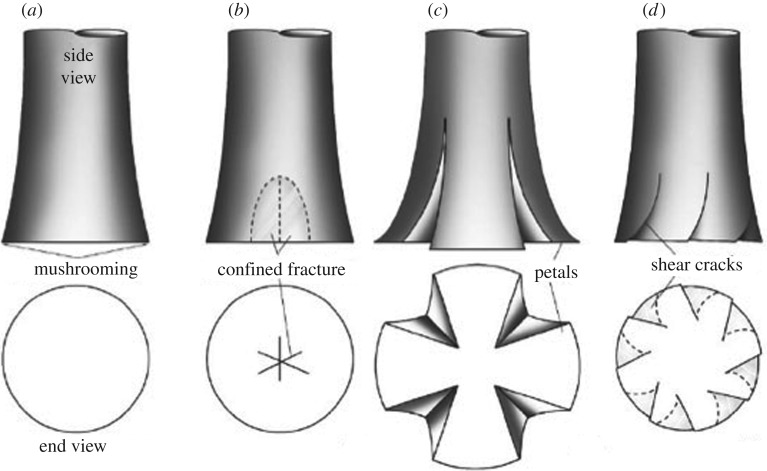
Schematic of classic failure modes observed during a ductile to brittle transition in fracture behaviour in Taylor cylinder impact specimens: (*a*) mushrooming, (*b*) confined fracture, (*c*) petalling and (*d*) shear cracking (adapted from [9]).

The outcomes of Taylor tests on thermoplastics are dependent on the operating mechanisms at different length scales within the microstructure with response conditioned by both the chain and mesoscale morphology of the material as it deforms (figure 1). The modern adaptation of the Taylor cylinder impact test is a fully integrated experiment that highlights macroscopic behaviour with origins reflecting a multiscale response [19]. There are distinct regions of deformation that are controlled by compression and viscoplastic flow followed by release and fracture. In the first moments, the shocked head of the cylinder, and only a central conical portion of it, experience high impact stress and shock-loading in uniaxial strain. Release of the cylinder that results in waves propagating in from the outer free surfaces relieves this shocked region and when these fronts meet down the central axis, dynamic tension damages the material and fails it along its core (figure 1*b*). Furthermore, the expanding footprint on the impact face will eventually fracture radially under increasing hoop stresses and cracks will propagate back towards the axis. These modes of surface damage at the highest amplitudes eventually form a series of struts, which bend outwards from a hinge approximately one diameter back from the impact face to accommodate the strain, eventually fracturing in some materials and shortening the cylinder by fracturing its first diameter (figure 1*c*). Of course, these comments describe a laboratory scale description of the mechanical processes failing the cylinder. At lower length scales the material is highly anisotropic, with local moduli varying by orders of magnitude. These scales are accessed over different periods of the loading as the following observations will illustrate. Thus multiaxial loading in the Taylor cylinder geometry, and the composite nature of the microstructure at the mesoscale, result in a range of observed operating mechanisms occurring in compression but also in tension, as the material fails on release. This complexity means that the test is a sophisticated validation experiment for material models, but great care must be taken to specify the quantities for comparison, given the range of operating mechanisms and length scales available for diagnosis. Indeed, one of the conclusions of this work illustrates that the observations made previously using this test to obtain macroscale measurements of response have neglected an internal failure mode, one which can only be observed using advanced methods such as high-speed imaging coupled with X-ray tomography.

## 2. Material and methods

**Figure 2.**
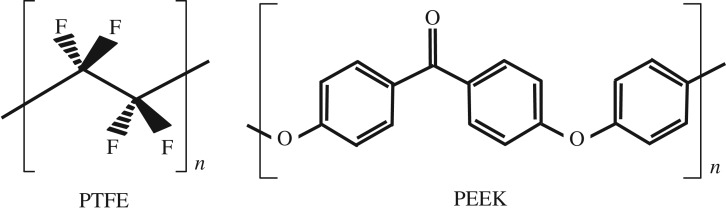
Monomer microstructures for the two polymers chosen (after [19]).

The complex behaviour probed in previous work on different classes of polymer suggested the two thermoplastics chosen for this study, with well-defined physical properties over a large stress and temperature range (figure 2).

The two thermoplastics chosen, PTFE and PEEK, illustrate the complexities of polymer response under dynamic loading. Illustrative material properties, some collected under shock conditions to cover the range of states accessed in the test, are collected in table 1. PTFE (tradename Teflon), is a widely employed fluoropolymer found in many engineering applications. The material is semicrystalline under ambient conditions with linear chains adopting several, complex phases within crystalline domains near room temperature and ambient pressure [21–24]. Under these laboratory conditions, a pressure-induced phase transition has been reported in PTFE at 0.50–0.65 GPa (phase II–III). Phase II PTFE consists of a helical conformation with a 13 atom repeat unit and a well-ordered hexagonal packing of the helical chains, while in phase III the helical conformation gives way to a planar zigzag and the chain packing takes on an orthorhombic or monoclinic lattice structure. The phase transition results in a 13% local volume decrease within the crystalline domains and a considerable reduction in compressibility. PTFE also exhibits two atmospheric pressure, crystalline transitions at 19°C and 30°C. PEEK is a semicrystalline thermoplastic with excellent mechanical and chemical resistance properties that are retained to high temperatures. The processing conditions used to mould PEEK can influence the crystallinity, and hence mechanical properties, under impact. It is a two-phase semicrystalline polymer, consisting of amorphous and crystalline domains. It has been shown in previous work that the mechanical properties of PEEK plastics are influenced by the degree of crystallinity. Hamdan & Swallowe reported an increase in crystallinity of samples deformed by large strains under adiabatic conditions [11,25]. Conversely, Rae and co-workers [12,13] showed a decrease in crystallinity of all samples that were deformed to large strains. However, it is known that adiabatic heating, associated with the impact process can induce rapid crystallization of PEEK at temperatures above the glass transition [12].

**Table 1.** Physical properties of PTFE and PEEK. *ρ*, density; *c*_L_, the longitudinal wavespeed; *c*_S_, the shear wavespeed; *c*_0_, the bulk sound speed and *S*, the shock constant for the material (see [19–21]).

name	*ρ* (g cm^−3^)	*c*_L_ (mm µs^−1^)	*c*_S_ mm µs^−1^)	*c*_0_ (mm µs^−1^)	*S*
polyetheretherketone (PEEK)	1.30	2.47	1.06	2.52	1.71
Teflon Carter & Marsh (PTFE high P)	2.15	1.29	0.71	1.84	1.71
Teflon low P regime (PTFE low P)	2.15	1.23	0.41	1.14	2.43

## 3. Experiment and imaging

Taylor cylinder impact tests were performed on 7.6 mm diameter, 38 mm length (length/diameter *L*/*D* = 5:1) PTFE and PEEK cylinders. The cylinders were fired from a single stage gas gun onto a hardened steel anvil in air. Molybdenum disulfide grease was used on the surface of the anvil prior to each impact to ensure that the coefficient of friction between anvil and cylinder was kept as close to zero as practicable. A digital high-speed camera, operating at approximately 120 000 frames per second (8 µs interframe time; IFT) and with a 1 µs per frame exposure time, was used to record the events.

**Figure 3.**
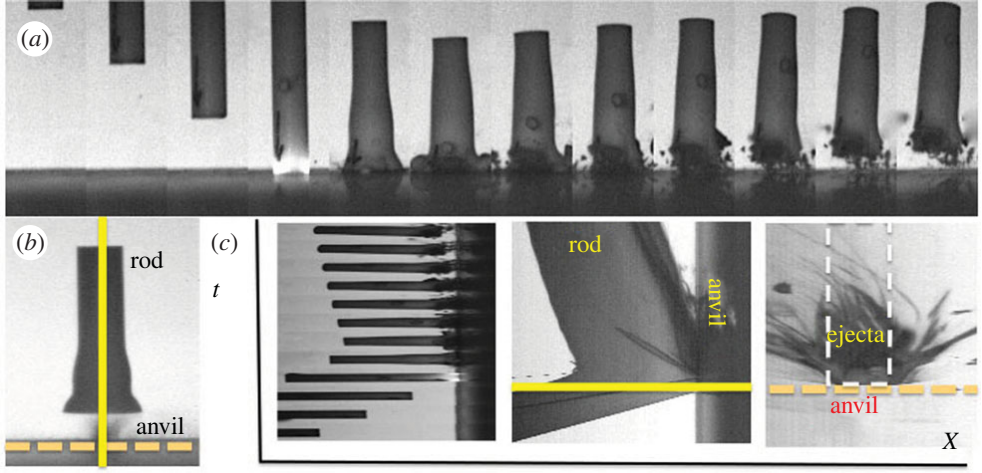
(*a*) High-speed images gained from an impact of PTFE onto steel anvil at 122 m s^−1^. IFT for this sequence 33 µs. (*b*) Image of a frame with (solid) vertical and (dotted) horizontal streak axes to diagnose impact. (*c*) Streak images of impact. Left, a stacked sequence of cylinders composed of the sequence in (*a*); horizontally, centre, vertical streak of same impact; right, surface ejecta from horizontal streak axis on anvil impact surface. Time runs vertically and distance horizontally in each part of (*c*). (Online version in colour.)

In all the tests, high-speed imaging was used to record quantitative macroscopic deformation. The camera simultaneously recorded both framing and streak images down both an impact axis and in the plane of the impact on the anvil surface. The images are presented conventionally, with the spatial axis running horizontally and the temporal running vertically. A typical framing sequence for impact of a PTFE cylinder is shown in figure 3*a* and the two streak axes are illustrated in figure 3*b*. Figure 3*c* shows two streak records for the impact illustrating cylinder impact onto the anvil and ejecta motion from the surface. Time runs up the page in this representation and the slope of interfaces and particle tracks allows a direct measure of velocity in what follows.

The Taylor cylinder impact test samples were soft recovered after impact and then individually scanned using phase contrast X-ray tomography at the Diamond Light Source (http://www.diamond.ac.uk/Beamlines/Materials/I13.html.). The I13-2 Diamond-Manchester beamline generated X-rays with an intensity of 2 × 10^8^ photons per second and a pink beam spectrum with average energy around 22 keV. Precision optics viewing a scintillation screen captured 2661 individual digitalized radiographs per scan. Multiple X-ray views through the sample were recorded as it was rotated about its long axis, and a three-dimensional volume was reconstructed via a filtered back projection algorithm, allowing digital, two-dimensional cross-sectioning [26]. The images were processed, and three-dimensional visualization software (Avizo v. 9.0) was used to create full three-dimensional renderings of the recovered samples. The voxel size for each scan was 3.6 µm for these data.

## 4. Dynamic deformation

**Figure 4.**
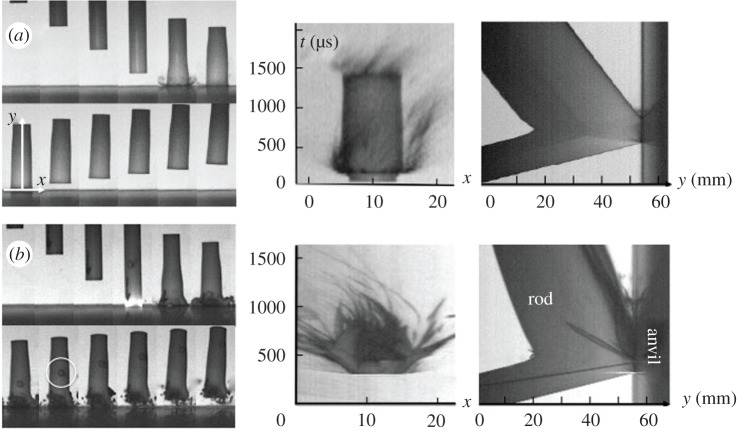
Taylor cylinder impact sequences (left) and two axes of streak (right). The IFT was 33 µs for each of the sequences shown in the figure. (*a*) PTFE 91 m s^−1^ and (*b*) PTFE 122 m s^−1^. Seventh frame of (*a*) shows two streak axes, *x* and *y*.

**Figure 5.**
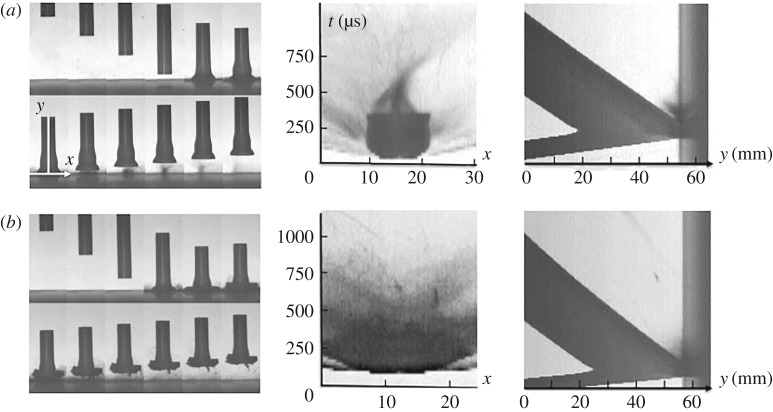
Taylor cylinder impact sequences (left) and two axes of streak (right) on (*a*) PEEK 313 m s^−1^ and (*b*) PEEK 391 m s^−1^. Seventh frame of (*a*) shows two streak axes, *x* and *y*.

Figure 4 shows two illustrative sequences and analyses taken from high-speed photography of impacts on PTFE. Impacts were at 91 m s^−1^ and 122 m s^−1^ with PTFE in these cases. Shots were conducted at higher and lower velocities than those shown here in figures 4 and 5 but these serve to illustrate a key change in behaviour observed in the two polymers.

This new work on Taylor cylinder impact using PTFE cylinders builds on previous work by us and focuses particularly on the compressive response and phase transformation that occurs in the impact region of the cylinder [27,28]. In this work, the progressive accrual of damage and tensile failure is investigated. Figure 4*a*,*b* shows the impact, compression and tensile damage above and below the threshold at which cracks eventually detach material from the cylinder in the first diameter of the cylinder. At the lower speed (in figure 4*a*), impact can be seen in the fifth frame where a jet of expelled grease can be seen jetting out from the lubricated surface. In last frames, compression can be seen as an inelastic front preceding mushrooming which passes down the cylinder. This is observed in two volumes; one in the impact zone and one that extends back at this impact velocity to almost the rear face of the cylinder. The streak images to the right of the sequence show deformation progressing in the *x-* and *y-*directions (defined in frame 7 of the sequence). The surface *x* streak on the anvil surface show fast expansion of a surface zone to an almost constant new compressed diameter. This is retained as the cylinder rebounds off the surface. There is an expansion of 30% in cylinder diameter on impact at this speed. Grey streaks surrounding this region, correspond to grease ejected from the lubricated impact face. The axial *y* streak shows the cylinder entering from the left, stationary on the surface of the anvil as the cylinder compresses, and then rebounding as it exits. The slopes of these lines correspond to the axial velocities of impact and rebound. The average rebound speed of the cylinder is 33 m s^−1^ that is approximately 36% of the impact speed. Interactions within the cylinder can be seen as waves arrive at free surfaces decelerating and then accelerating it after rebound. These appear as discontinuities in the slopes of the interface lines in the streak photograph. The puff of grease from under the impact face can be observed as a grey jet as the cylinder leaves the surface. Note that the slopes of the trajectories of the rear surface entering and leaving are not the same at later times. Plastic work has been done in compressing the microstructure and the cylinder leaves at a lower velocity than it enters. The *y–t* streak image shows the average rebound velocity to be approximately 7 m s^−1^, which is only 6% of the input speed for the 122 m s^−1^ experiment. It is clear that in this case there is significant energy going into the phase transition and subsequent fracture, as well as plastic deformation.

The higher velocity shot in figure 4*b* exceeds the speed required for quasi-brittle fracture of the impact zone and in this case a shortened cylinder exits the anvil. In frame 4 of the sequence a flash occurs, believed to be fractoemission from the cracking polymer. In later frames, the compression and radial expansion of the cylinder continues as in figure 4*a*, but more pronounced local surface damage is seen. Eventually, this zone collapses and fragments of material are ejected across the surface. This process is seen graphically in the *x* streak where failure and emission of polymer particles can be seen immediately after impact. The axial *y* streak shows collapse onto the surface and rebound at a much slower velocity (approx. 7 m s^–1^) to that seen in figure 4*a*. An interesting feature is a dark line seen on the figure and across the cylinder coming in and reflecting back after impact. It corresponds to the image of a circle marked onto the external surface of the cylinder (and circled in frame 8 of the sequence). It was included to detect rotation and shows indeed that there is slight deflection on this shot during launch. This is not believed to affect the compression seen but may in other materials account for the presence of shear features in the surface zone (figure 1*d*).

Figure 5 shows two sequences from a series conducted on the polymer PEEK. They again cross a boundary in surface failure behaviour for the material in this test. The PEEK impact in figure 5*a* shows features typical of those seen in the low velocity behaviour of the polymer and is qualitative similarity with that seen for PTFE above. The cylinder is deformed on the anvil and plastic deformation occurs within the polymer but no fracture of the expanding edge occurs and no mass is lost. Frame 7 of the sequence has two axes *x-* and *y-*superimposed on the image. These represent two streak axes for the impact and the streak images are shown to the right of the framing sequence. The central *x–t* shows the intrusion of the cylinder onto anvil and it spreading for the contact time until release allows it to return with a greater impact face diameter. In this case, the diameter is 83% greater than before impact. The *y–t* (furthest right) shows the cylinder entering, impact onto the surface, return of the plastic wave from impact and rebound of the cylinder back off the surface in its shortened and deformed state. It rebounds at a speed of approximately 70 m s^−1^ which is 22% of the impact speed. Figure 5*b* shows a further impact on PEEK but at a velocity increased by approximately 80 m s^−1^ over that in figure 5*a* and now sufficient to rupture, damage and involute the expanding impact zone. The *x–t* streak shows much greater damage than was evident for the slower case, while the *y*–*t* shows a shortened cylinder but similar form to the first. The average rebound speed is close to the same as that in figure 5*a*, yet involution of the cylinder face has occurred in this case. The *y–t* streak images show the rebound velocity to be 22% and 18% of the input velocity for the 313 m s^−1^ and 391 m s^−1^ case, respectively. In the higher velocity case, in particular, the cylinder is slowing as time progresses and the impact face thus shows a convex form. This divergence of the streak for the two ends of the cylinder after the rebound suggests viscoelastic relaxation on the time frame of the test.

It will be seen from these macroscopic observations that the two materials behave in a similar manner in compression but fail very differently in tension. A longer dwell time in contact with the anvil is observed for PTFE. Thus in this material in particular there appears to be a ductile–brittle transition in behaviour, which has been noted by others in previous work [27,28].

## 5. Tomographs and reconstructed damage

**Figure 6.**
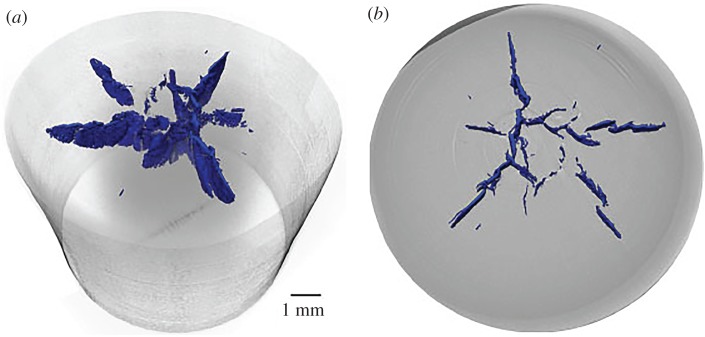
Damage in a recovered cylinder of PTFE impacted at 108 m s^−1^. The solid (blue online) regions show the fracture surfaces within the polymer introduced by impact. (*a*) A three-dimensional reconstruction of the fracture surface and (*b*) a section at 1 mm from the target surface. At this plane the fractured regions are clearest. Voxel size for scan 3.6 µm. (Online version in colour.)

Figure 6 shows tomographs for damage in PTFE below the fracture transition observed macroscopically. In all cases, the deformed cylinder outline is shown in grey while the crack surface has been highlighted in blue (online). Figure 6*a* shows a reconstruction of the fracture surface. It is clear that cracks extending from the impact face travel radially outwards and extend back in the shocked region of the cylinder. The slice in figure 6*b* shows a central region from which fracture appears to originate. In this case, the highest pressures might be expected to occur here and a phase transition will occur if the threshold pressure were reached. The measured shock parameters for PTFE and PEEK in table 1 can be substituted into the expression for stress, *σ*, on the impact face

5.1 $$\sigma =\rho c_{0}\text{v},$$

where *v* is the impact velocity. At these speeds a shock pressure of approximately 0.3 GPa will be induced, calculated using the available measured, Hugoniot data [21].

Under laboratory conditions at room temperature, a pressure-induced phase transition has been reported in PTFE at 0.50–0.65 GPa (phase II–III) so that with the shear components aiding rearrangement, this region is likely to correspond to a transformed and stronger phase [21]. Certainly, the observed fracture morphology suggests this to be the case.

**Figure 7.**
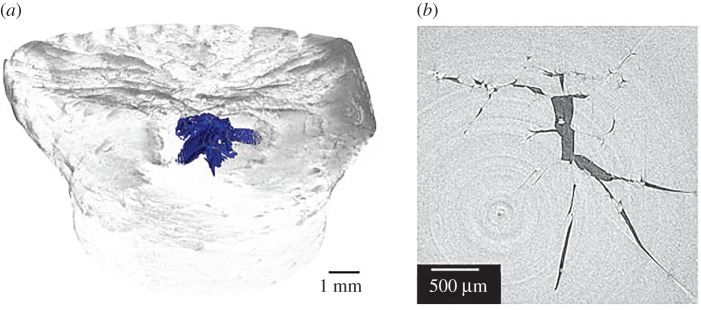
Recovered PEEK cylinder impacted at 288 m s^−1^. (*a*) Blue fracture surface and (*b*) section showing fracture morphology away from the impact face. Voxel size for scan 3.6 µm. (Online version in colour.)

By contrast, figure 7 shows equivalent scans for PEEK in the low-pressure regime. In this case, the failure can be seen to lie below the impact face within the material. Further, the impact face itself is of concave form after recovery as it exits from the anvil. The (blue) fracture zone, however, is formed of cracks propagating out from the central axis of the cylinder in this case as opposed to the PTFE where failure started away from this transformed region. The fractured region is shown in figure 7*b* and can be seen to consist of cracks opened from the central region and then moving outwards, and in one case bifurcating, into the relieved region at the impact face. In local regions, there is evidence of torn material around material defects opening in small cracked regions consistent with damage starting from a macroscopic zone loaded in tension, with local failure initiated at flaws within the polymer.

**Figure 8.**
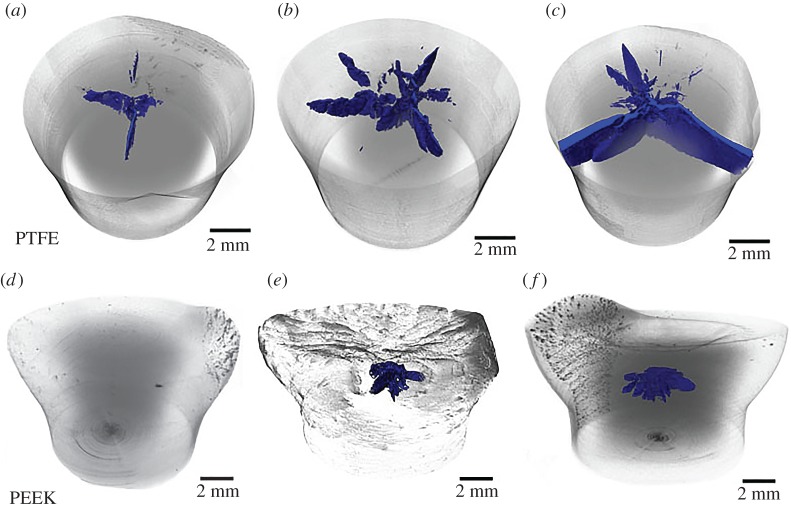
PTFE and PEEK tomographs; fracture surfaces highlighted in blue (online). Impact velocity for each recovered target PTFE (*a*) 91, (*b*) 108, (*c*) 115 m s^−1^; PEEK (*d*) 212, (*e*) 288, (*f*) 313 m s^−1^. Voxel size for scan 3.6 µm. Tomographic data of fracture voids provided as the electronic supplementary material. (Online version in colour.)

Figure 8 shows three-dimensional renderings of PTFE (figure 8*a*–*c*) and PEEK (figure 8*d*–*f*) recovered cylinders across the velocity range and shown together. There are three images for each polymer showing evolution of damage with increasing velocity with the impact face at the top of the figure. Note that the equivalent regimes for onset and propagation of damage are of order 100 m s^−1^ in the case of PTFE, but 300 m s^−1^ for PEEK, reflecting the different high-rate strengths of the two materials. For both polymers, there is an increase in fracture surface area with increasing impact speed (excepting the PEEK cylinder fired at 212 m s^−1^ that showed no internal fracture). In both polymers, the maximum tensile stress was applied on the impact axis where radial release interacted during the shock-loading phase. This led to fracture when the material had not undergone phase transformation. Quantitative analysis of these fractured zones is presented below.

For all the PTFE cylinders (figure 8*a*–*c*), cracks extend and eventually penetrate the impact face. Radially, the number of cracks and the extent of damage increases with velocity, and by 115 m s^−1^ cracks have reached the cylinder circumference (figure 8*c*). Beyond this speed individual segments were levered at internal hinges, before fracturing and then being expelled, from the surface region as seen in the lower sequence of figure 4. There is also more radial crack bifurcation seen with increasing impact speed. In the shock region (loaded in one dimension in a conical geometry), as commented above there is a central segment where failure is restricted that corresponds to the region identified previously as a transformed phase [21]. It is possible that a shear surface, created between transformed and untransformed material, nucleates fracture in the polymer.

Fracture in the PEEK samples was seen to be of a different morphological nature to that of the PTFE samples; being fully contained within the cylinder and not extending to the impact face or the cylinder circumference in the targets shown. The cracks were initiated down the impact axis and bifurcated as they extended radially outwards. Again the number of bifurcations present appeared to increase with greater velocity. It will be seen that the fractures are not found in the region near the impact face; the damage region is encapsulated behind a transformed surface zone (figure 8*e*,*f*). The surface on recovered targets is concave as seen with previous studies and the damage is localized away from here in all cases [29].

The quantitative data measured through analysis of the tomograms clearly illustrates the differences in fracture behaviour and morphology between the two polymers (table 2). In the case of PTFE, the increasing velocity affects the integrated crack surface area, which increases at the higher velocities considered here, and also plays an important role on the evolution of damage and its morphology. In particular, the primary crack detected for the highest velocity considered (115 m s^−1^) shows a morphology consistent with that observed at the lower velocities, where the damage originates from the central core of the impact cylinder and propagates towards the edges (figure 8*a*,*c*). In these cases there are more fragmented and discontinuous cracks towards the edges (figures 6 and 8*b*). The volume enclosed within the cracked region increases significantly and indeed jumps at the highest velocity analysed in PTFE as the cracks reach the outer free surface.

**Table 2.** Measured fracture dimensions in the damaged regions. The resolutions in the analysis are the same for each sample so that systematic bias is the same for each material. The absolute error on each measurement is within ±1 of the last significant figure quoted.

sample	impact velocity (m s^−1^)	crack area (mm^2^)	crack volume (mm^3^)	crack length (mm)	location
PTFE 1	91	14.6	0.19	1.50	from impacted surface
PTFE 2	108	58.6	0.86	2.25	from impacted surface
PTFE 3	115	89.6	4.16	3	from impacted surface
PEEK 1	212	0	0	n.a.	n.a.
PEEK 2	288	24.7	0.32	1.95	0.75 mm below impacted surface
PEEK 3	313	36.0	0.43	1.95	1.05 mm below impacted surface

In the case of PEEK, increasing impact velocity is accompanied by an increase in crack area, but not as significantly as in the case of PTFE. Cracks are initiated at similar locations and at a similar crack length (depth) for impact velocities of 288 and 313 m s^−1^, respectively. The change in behaviour between these two velocities is represented by the increasing crack area with higher velocities (extending towards the edges), but more interestingly the increase of velocity is accompanied by a shift of the damage zone with respect to the deformed impacted surface (0.75 mm below for a velocity of 288 m s^−1^ as opposed to 1.05 mm below for the impacted velocity of 313 m s^−1^). It will be noted that the relative crack volumes for PTFE and PEEK are very different, indicative of differing fracture behaviours. The PEEK shows tendrils bridging the crack leading to an opening displacement that is small showing its resistance to fracture relative to PTFE.

## 6. Discussion

The two polymers respond in a very different manner to the applied loading pulses. The behaviours observed are conditioned by the response of the material in compression, release and then further tension. There are several phases of loading acting on different timescales and affecting different zones within the cylinder that will be described and explained in what follows. Immediate deformation at the impact face in the first moments drives a shock front back into the target and loads a zone, which is released from the free surfaces as they expand laterally. In PTFE, this zone is placed at pressures above the phase II–III transformation pressure (as calculated using equation (5.1) above) and it is this that preconditions material for tensile failure at later times. However, the surface itself remains planar as the cylinder rebounds and recovers. The PEEK cylinder deforms on the flat surface but on rebound recovers, forming the concave surface noted in the imaging section above. Further, a damage zone is only found some way back into the target and then only at later times. There is a zone of greater strength immediately behind the impact face in this material. Previous experiments have observed a similar region with different properties in impacted PEEK that has undergone large strains [30].

The two materials show differing crack morphologies in both cases nucleated on the impact axis but, in the case of PEEK, confined to a zone away from the impact face. In PTFE radial cracks propagate towards the edge of the sample and when they reach a free surface will open up slices that for struts that will bend back and eventually fracture as velocity increases to accommodate the applied strain.

There is a more complex petalling in the case of PEEK. In this case bridging ligaments are more visible along the radial direction (towards the edges), while cracks are more continuous along the crack depth (direction perpendicular to the impacted surface). Further the location of the damage lies below the impact surface in the case of PEEK; cracks are found connected with the impacted surface for PTFE, while damage in PEEK is 0.75 mm and 1 mm below the impacted surface. Finally, there is a smaller crack surface area measured in the case of PEEK compared with the damage quantified in the PTFE targets.

The dynamic phase transition in PTFE leaves a zone of differing properties which has a dramatic effect upon the increasing crack surface area measured from the tomograms. An increase of approximately 20 m s^−1^ results in a large increase in crack length radially as hoop stresses act. There are in this case a limited number of bridging ligaments and high crack opening displacements. Increasing velocity in PEEK (a difference of approx. 30 m s^−1^) generates a smaller crack surface than for PTFE. In this case, there is no change in crack depth and an increase of radial extension of the crack. The change to a higher velocity shifts the crack initiation site a further 0.3 mm below the impacted surface for this small increment in velocity. As seen in table 1 PTFE in each phase shows different wavespeeds which also affects fracture propagation.

**Figure 9.**
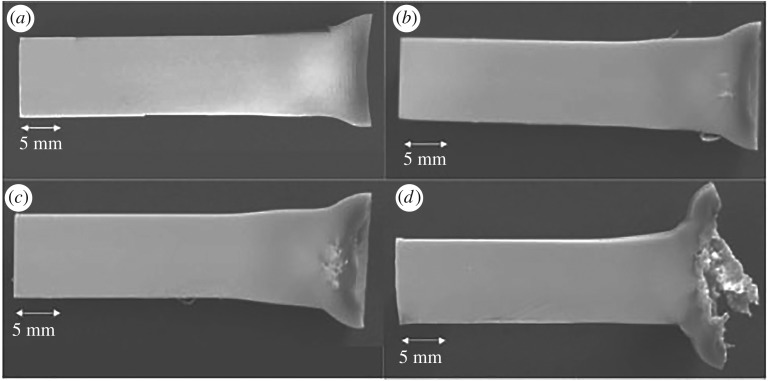
Recovered PEEK cylinders of 10 mm diameter [29,30]. Sectioned PEEK Taylor cylinders after impact. Impact velocities: (*a*) 247 m s^–1^; (*b*) 276 m s^–1^; (*c*) 303 m s^–1^ and (*d*) 349 m s^–1^ (adapted from [29]).

Figure 9 shows sections of four PEEK Taylor cylinders after impact over a similar stress range. As seen above, the loaded impact face of each recovered cylinder has a strongly concave nature, showing that relaxation has occurred after impact. Discoloration immediately under the impact face can be seen in all cases and in this region material is significantly darker than the bulk of the cylinder. It is concluded that the polymer is undergoing rapid deformation as the impact face flows across the anvil. High pressures, temperatures and large lateral strains are found that are not present in other parts of the cylinder specimen. In other work, the changes in crystallinity at the ends of the Taylor cylinder impact samples were measured by differential scanning calorimetry and accurate density measurement techniques as a function of impact velocity [13]. It was found that the change in colour corresponds to regions where the percentage crystallinity of PEEK is decreased relative to the original spherulite morphology in the as-received sample [13]. In figure 9*c*,*d*, tensile damage on the central axis of the cylinder can be observed analogous to that seen using tomography. The combined recovered targets indicate the complex cycling of the mesoscale microstructure under load and the recovery that occurs under the extreme conditions at the impact face, driving irreversible changes in material properties that result from such loading. This has been quantified here for the first time using quantitative tomography on the recovered cylinders.

Clearly, the strength of polymers is controlled by electronic and steric interactions that act in unison to define the regime that these materials inhabit during dynamic loading. At low pressures, the molecular spacing and conformation is key in defining the development of the material's strength as compression increases. However, in all the cases investigated, polymers show increasing shear strength with pressure as the open microstructure rearranges as it approaches full density [19, p. 397]. The weak van der Waals interchain forces are easily overcome by even modest compression. Resulting densification and stiffening due to interchain repulsion means that an elastic region comparable with that seen in the undeformed state does not truly exist. By contrast, a regime is developed within which microstructure rearranges to accommodate the strain after initial fast densification, and this results in increased strength within the polymer. The fluorine encasement of each polymer chain reduces the material strength in PTFE relative to stronger van der Waals bonding and increased steric hindrance found in PEEK. The tomographs of figure 8 show the evolution and completion of tensile failure in the first diameter of the cylinder striking the anvil. The radial tensile failure in the two cases results from release of the compressive shock state. Applying conservation equations (equation (5.1)) shows impact stresses of order 0.3 GPa for PTFE. Those in PEEK are almost four times higher at 1.1 GPa at higher impact speeds before complete material failure was observed (see shock data in table 1 and [19]). These results explain the apparent anomaly observed in the data collected previously on these materials where the transition from a damaged but intact state to a damaged and fractured cylinder was interpreted as a ductile–brittle transition [27]. This is supported by recovery, where the cylinder length becomes markedly shortened over a small velocity range corresponding to the onset of fracture and development of petals that hinge outward to absorb the forward momentum of the cylinder and accommodate strain. It is of course possible the increased velocities and resulting compressions are accessing a new range of defects as speed increases. Thus, stochastic processes may be responsible for the increased damage observed. However, our new work shows for the first time that there is a continually evolving internal damage state in the region before failure.

PTFE has also been shown to have a low-pressure phase transition at 0.65 GPa and this occurs in high velocity shots in this series of experiments [21]. Observations have revealed changed mechanical properties after transformation, including altered moduli and increases in crystallinity. There is evidence of some early transformed regions in some grains in these experiments, but the failure of the cylinder occurs before there is a homogeneous transition across the whole volume [27]. In PEEK the loaded end of each recovered cylinder has a strongly concave nature, showing that considerable relaxation has occurred after impact. Discoloration immediately under the impact face was seen previously and it is shown there that fracture always propagates behind this region [12,29]. This change in colour corresponds to regions where the percentage crystallinity of PEEK is decreased relative to the original spherulite morphology in the as-received sample. This shows the complex cycling of the mesoscale microstructure under load and the recovery that occurs under the extreme conditions at the impact face that drives the irreversible changes in material properties that result. This work has added to the previous macroscale experiments with increased understanding of the development of damage only seen with the use of high-resolution tomographic techniques. This changes micromechanics considerations of loading and failure and should lead to new physically based models for polymer failure at high strain rates.

## 7. Conclusion

This work has shown the great utility of using this simple, cylindrical geometry in probing the compression and failure of plastics under variable strain-rate loading. The Taylor cylinder impact test has been a useful tool for elucidating deformation mechanisms in polymers and composites. The macroscale observations presented here are consistent with previous studies. However, this work has shown that the behaviours deduced from macroscopic high-speed imaging, or sample recovery and examination of external surfaces alone, are insufficient to describe the complex damage and compression response of this class of materials. This methodology for the tests presented here and these recovery measurements illustrated, offer a quantitative measurement of damage for this macroscopic biaxial stress state that correlates with the identification of new mechanisms operating under load.

The shocked region only compresses a surface zone that extends back one diameter from the impact face. Within this region the outer radius of the impact footprint flows outwards and subsequently fails under hoop stresses at the periphery while the interactions of radial releases from the free surfaces at the central axis initiate fractures travelling outwards from the core. PTFE has a network of confined cracks propagating from the impact face below the phase transition not previously reported. These cracks from the central region fail the cylinder before those from the expanding footprint become significant. PEEK exhibits ductile deformation, darkening (consistent with a reduction in crystallinity) and damage behind the impact surface.

The combination of an idealized Taylor cylinder impact loading geometry with X-ray tomographic imaging has shown that one may obtain a higher fidelity view of damage in three dimension than has been seen before. This is part of an ongoing effort to extend use of the test from a validation and verification of constitutive models to one in which one identifies operating mechanisms and derives constitutive descriptions instead. This dual approach allows us to uniquely identify and quantify failure in this important class of materials. Mapping behaviour across a range of materials will open new doors to understanding dynamic material failure.

## Supplementary Material

Tomographic image data representing the fracture voids as shown in Figure 8

## Data accessibility

Tomographic image data representing the fracture voids in the PTFE and PEEK samples shown in figure 8 are available as the electronic supplementary material. These have been binned by a factor of three along each axis to reduce the file size.
